# The role of neutrophil PD-L1 expression in acute exacerbation of COPD

**DOI:** 10.1371/journal.pone.0355281

**Published:** 2026-07-30

**Authors:** Wei Sun, Liyu Zheng, Haibin Li, Li An, Jing Wang, Chao Ren

**Affiliations:** 1 Department of Respiratory and Critical Care Medicine, Beijing Institute of Respiratory Medicine and Beijing Chao-Yang Hospital, Capital Medical University, Beijing, China; 2 Translational Medicine Research Center, Medical Innovation Research Department of the Chinese PLA General Hospital, Beijing, China; 3 Medical Research Center, Beijing Chao-Yang Hospital, Capital Medical University, Beijing, China; Stanford University School of Medicine, UNITED STATES OF AMERICA

## Abstract

**Background and objective:**

The elevation of neutrophil-lymphocyte ratio (NLR) has been shown to be critically involved in unfavorable outcomes among patients with chronic obstructive pulmonary disease (COPD). The mechanism underlying NLR changes during COPD progression remains unclear. This study evaluates the association between neutrophil PD-L1 expression and acute exacerbation of AECOPD and examines its correlation with the NLR.

**Methods:**

In this prospective observational study from June 1 to December 30 2024, we enrolled 14 patients with acute exacerbation, 27 with stable conditions, and 9 healthy control subjects. Blood samples were collected for neutrophil isolation, and the CD15^+^CD274^+^ neutrophil percentage was measured using flow cytometry. We also recorded and analyzed smoking history, pulmonary function test results, and blood routine test data.

**Results:**

The percentage of CD15^+^CD274^+^ neutrophils was notably higher in patients with COPD compared to healthy controls and was even more elevated in those experiencing acute exacerbations of COPD (AECOPD) than in patients with stable COPD. This increased percentage was positively correlated with NLR but negatively correlated with FEV_1_% (FEV_1_ percentage of the predicted value). The AUC of CD15^+^CD274^+^ neutrophil percentage for predicting AECOPD was 0.894 with 95% CI 0.8–0.989 and a ratio above 3.273 (odds ratio 1.386; 95% CI 1.016–1.891; P = 0.039) independently associated with AECOPD.

**Conclusion:**

Peripheral blood neutrophil PD-L1 expression was elevated in AECOPD and independently associated with exacerbation status in this pilot cohort. Larger, multi-center studies with mechanistic validation are required to determine its clinical utility.

## Introduction

Chronic obstructive pulmonary disease (COPD) is associated with a chronic inflammatory response which is marked by an elevated presence of various immune cells and disturbed responses [1]. The loss of balance between innate and adaptive immune response, such as the ratio of neutrophil-to-lymphocyte (NLR), is one of the major signs of immune dysfunction and critically involved in the development of various diseases [2,3]. Notably, patients with COPD show marked activation of neutrophils and impaired lymphocytes function [4]. The NLR has been well investigated in numerous studies as a predictive marker for disease severity [5], hospitalization [6], and mortality [7] in patients with COPD. However, the underlying mechanism responsible for this immune dysregulation remains poorly elucidated.

Previous research has identified that the interaction between programmed death receptor 1 (PD-1) and its ligand (PD-L1) is an important inhibitory immune checkpoint. Microbes and malignant cells exploit this pathway to suppress antimicrobial and antitumor immunity, perpetuating chronic infection and tumor progression. CD15 is a neutrophil surface marker, while CD274 encodes PD-L1; accordingly, CD15⁺ CD274⁺ neutrophils represent PD-L1-expressing neutrophil subsets. The expression of PD-L1 can be modulated by a variety of factors, including pharmacological agents [8], persistent infections [9], and hypoxic conditions [10]. Among patients with sepsis, there is an upregulation of PD-L1 expression on neutrophils, which induces lymphocyte apoptosis through direct interaction [11]. Prior studies in patients with COPD have reported reduced PD-L1 expression on macrophages and dendritic cells [12,13]. PD-L1 is a key immune checkpoint, and its abnormal levels indicate immune dysfunction. To date, no studies have examined whether neutrophil PD-L1 expression correlates with the NLR or exacerbation risk in patients with COPD. Our prior research [14] indicates a significant association between the elevated NLR and adverse clinical outcomes in patients with AECOPD (acute exacerbation of COPD). This study aims to evaluate the potential role of neutrophil PD-L1 as a risk factor for AECOPD and to investigate the correlation between neutrophil PD-L1 and the NLR.

## Materials and methods

Verbal consent was obtained in person by qualified clinicians, who provided a comprehensive explanation of the study’s objectives, data utilization, potential benefits, non-invasive risks, and the rights of participants, including the right to withdraw without any repercussions. All consent conversations were witnessed by an independent staff member to ensure participants comprehended the information and voluntarily consented to participate. The study protocol, consent process, rationale for ethical exemption, and record-keeping procedures were approved by the Ethics Committee of Beijing Chao-Yang Hospital(2024-KE-414-1).

This analysis comprises data from a total of 41 patients with confirmed COPD, including stable COPD patients (n = 27) and those with acute exacerbation (n = 14), and a total of 9 healthy controls was also included between Jun.1 2024 and Dec.30 2024. The subjects participating in this study self-reported as Asian Han Chinese.

The detailed inclusion criteria were listed as follows: age ≥ 40 years old; COPD is defined as heterogeneous lung condition characterized by chronic respiratory symptoms (dyspnea, cough, expectoration and/or exacerbations) due to abnormalities of the airways (bronchitis, bronchiolitis) and/or alveoli (emphysema) that cause persistent, often progressive, airflow obstruction(post-bronchodilator FEV_1_/FVC[Forced Expiratory Volume in the 1st second/Forced Vital Capacity] ratio less than 70%) [15]. AECOPD is defined as an event characterized by increased dyspnea and/or cough and sputum that worsen in <14 days which may be accompanied by tachypnea or tachycardia [16] (Patients presenting with AECOPD did not complete in-study pulmonary function testing; accordingly, we used results from their most recent prior spirometry assessments). We enrolled only patients with moderate AECOPD managed in the outpatient setting with short-acting bronchodilators, oral corticosteroids, and oral antibiotics. The patients with COPD in this study received inhaler triple therapy. Patients with stable COPD experienced no exacerbation for a minimum duration of three months.

Exclusion criteria were as follows: incomplete clinical data; presence of other severe pulmonary diseases (e.g., asthma, bronchiectasis); end-stage chronic organ dysfunction or active malignancy, patients received immune checkpoint inhibitors, prolonged (>14-day) systemic glucocorticoid or immunosuppressant therapy, patients during pregnancy or lactation.

Patients’ baseline characteristics were recorded, including gender, age, smoking history and comorbidities. The control group was matched with COPD patients based on age, gender, comorbidities, and smoking status. The values of FEV_1_% (FEV1 percentage of the predicted value), FEV_1_/FVC and the presence of respiratory failure were recorded. The values of C-reactive protein (CRP) and routine blood parameters were obtained. NLR was calculated by the ratio of the total number of neutrophils to the total number of lymphocytes.

### Blood neutrophil isolation and flow cytometry

The samples were mixed with an isolation buffer in a 1:1 ratio and centrifuged at 600g for 30 minutes at room temperature. This separation yielded two distinct cellular fractions: mononuclear cells in the upper layer and granulocytes in the lower layer. Granulocyte pellets were transferred to a fresh tube, washed twice with phosphate-buffered saline (PBS), quantified, and resuspended in PBS at a concentration of 3 × 10^5^ cells per 100 μL for flow cytometric staining.

Briefly, raw flow cytometric events were first gated on a forward scatter (FSC) vs. side scatter (SSC) dot plot to exclude cellular debris, dead cells, and nonspecific particulate matter. Subsequently, doublet discrimination was performed by gating on FSC-area (FSC-A) versus FSC-height (FSC-H) to exclude cell aggregates and ensure analysis of singlet cells only. Within the singlet population, CD15⁺CD274⁺ cells were defined and quantified using appropriate fluorescence intensity thresholds. The flow cytometry was performed immediately following blood collection by magnetic sorting, ensuring cell viability exceeded 90%.

The neutrophils received antibody blocking by 1% bovine serum albumin (BSA, in PBS) for 1h at room temperature. Then these cells were stained with CD15 and CD274 by adding FITC-conjugated anti-human CD15 antibody as well as PE-conjugated anti-human CD274 antibody for 1h at room temperature. The percentage of CD15^+^CD274^+^ neutrophils was quantified via flow cytometry (BD Biosciences, USA).

This study aims to investigate the impact of PD-L1 expression on neutrophils in relation to the risk of AECOPD. Existing published data lack sufficient power to calculate an adequate sample size for this primary endpoint. Accordingly, we performed this pilot analysis to evaluate neutrophil PD-L1 as a predictive biomarker for AECOPD.

### Statistical analysis

To compare categorical variables, the Chi-square test was used. Normality of continuous variables was assessed via the Shapiro–Wilk test; normally distributed data were compared with independent Student’s t-tests, while non-normal data were analyzed with the Mann–Whitney U test. Pearson correlation was employed to evaluate the relationship between the proportion of CD15⁺CD274⁺ neutrophils and percent predicted FEV₁, and the results were displayed as correlation coefficients and P values. The partial correlation method assessed the relationship between CD15^+^CD274^+^ neutrophil and NLR, controlling for neutrophil count, and results were displayed as correlation coefficients and P values. Receiver-operating characteristic (ROC) curves were established to evaluate the ability of markers for predicting AECOPD. For each ROC curve, the optimal cutoff values, sensitivity, specificity, positive/negative predictive value, diagnostic accuracy, Youden’s index, area under curve (AUC), and 95% CI(confidence interval) were calculated. To determine if any markers were independently linked to acute exacerbation of COPD, we used logistic regression with a conditional forward stepwise model, yielding adjusted odds ratios (OR, 95% CI). Analyses were two-tailed, with significance set at P < 0.05.

## Results

**Fig 1:**
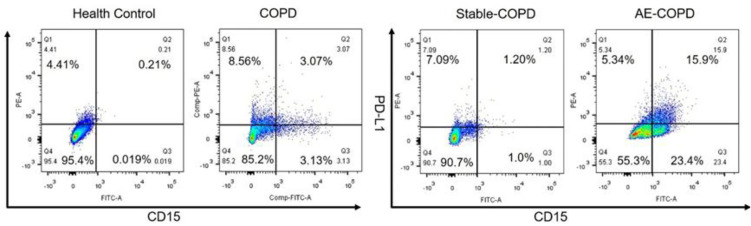
The percent of CD15^+^CD274^+^ cells by flow cytometry. **Abbreviations:** Q1: CD15^-^/PD-L1^+^; Q2: CD15^+^/PD-L1^+^; Q3: CD15^-^PD-L1^-^; Q4: CD15^+^PD-L1^-^.

There were no significant differences in terms of age, sex, hypertension, coronary heart disease, diabetes and smoking status between patients with COPD and control population. Besides, there were no significant differences in terms of CRP, blood routine and NLR between two groups. Importantly, the percentage of CD15^+^CD274^+^ neutrophil (Fig 1) was much higher in patients with COPD than that in control group (Table 1).

**Table 1:** Comparison between COPD population and healthy control subjects.

Characteristics	COPD (N = 41)	control subjects (N=9)	*P*
Age(years)	67 ± 10	66 ± 10	0.849
Sex (male,%)	36(87.8%)	7(77.8%)	0.595
Hypertension(n,%)	27(65.9%)	7(77.8%)	0.699
Coronary heart disease(n,%)	26(63.4%)	7(77.8%)	0.699
Diabetes(n,%)	31(75.6%)	6(66.7%)	0.679
Smoker(current/ex)	32(41%)	7(77.8%)	1
CRP (mg/L)	4.8(1.2 ~ 11)	5(2.65 ~ 5.75)	0.93
Leukocyte count(x10^9^/L)	6.9 ± 1.65	6 ± 1.46	0.136
Hemoglobin(g/L)	139.39 ± 19.59	132.89 ± 12.36	0.347
Platelet count(x10^9^/L)	209.34 ± 57.05	189.33 ± 39.91	0.324
Neutrophil count(x10^9^/L)	4.56 ± 1.53	3.79 ± 1.57	0.178
Lymphocytes count(x10^9^/L)	1.66 ± 0.63	1.55 ± 0.32	0.621
NLR	2.57(1.79 ~ 4.29)	2.48(1.39 ~ 3.19)	0.426
Eosinophils count(x10^9^/L)	0.14(0.07 ~ 0.24)	0.15(0.06 ~ 0.25)	0.8
CD15 ^+^ CD274 ^+^ Neutrophil (%)	3.67(1.08 ~ 9.87)	0.01(0 ~ 0.20)	<0.001

**Abbreviations:** COPD = chronic obstructive pulmonary disease; CRP = C-reactive protein; NLR = neutrophil/lymphocyte ratio.

We conducted a comparative analysis of the demographic characteristics and laboratory indices between patients experiencing AECOPD and those in a stable condition (Table 2). It showed that the age of AECOPD group was higher than that of stable-COPD. There were no differences in terms of sex, hypertension, coronary heart disease, diabetes and smoking history between two groups. The incidence of respiratory failure was significantly higher in AECOPD compared with the stable-COPD. FEV_1_/FVC and FEV_1_% were much lower in AECOPD compared with those in stable-COPD. The NLR in AECOPD group was elevated compared to the stable group; however, this difference did not achieve statistical significance, potentially attributable to the limited sample size. It was also showed that CRP were higher in AECOPD group than those in stable-COPD, and the levels of hemoglobin, platelet and lymphocytes counts were significantly lower in patients with AECOPD. There were no differences in terms of leukocyte and eosinophils count between AECOPD and stable-COPD patients. The percentage of CD15^+^CD274^+^ neutrophil was significantly higher in AECOPD patients than that in patients with stable-COPD.

**Table 2:** Comparison between AE-COPD patients and stable-COPD patients.

Characteristics	AE-COPD (N = 14)	Stable-COPD (N = 27)	*P*
Age(years)	72.36 ± 8.90	63.74 ± 8.83	0.005
Sex (male,%)	12(85.7%)	24(88.9%)	1
Hypertension (n, %)	8(57.1%)	19(70.4%)	0.494
Coronary heart disease(n,%)	7(50%)	19(70.4%)	0.306
Diabetes(n,%)	8(57.1%)	23(85.2%)	0.064
Smoker(current/ex)	11(78.6%)	21(77.8%)	1
Respiratory failure	7(50%)	0(0%)	<0.001
FEV1/FVC ratio	37(28 ~ 51.5)	60(42 ~ 64)	0.011
FEV1(%)	42.5(31.5 ~ 65)	68(50-86)	0.02
CRP (mg/L)	13.55 (8.63 ~ 22.5)	2.3 (1.1 ~ 5.8)	0.001
Leukocyte count(x10^9^/L)	6.34 ± 1.67	7.12 ± 1.60	0.118
Hemoglobin(g/L)	125.36 ± 21.56	146.67 ± 14.03	<0.001
Platelet count(x10^9^/L)	178 ± 52	226 ± 53	0.009
Neutrophil count(x10^9^/L)	4.56 ± 1.81	4.57 ± 1.40	0.983
Lymphocytes count(x10^9^/L)	1.21 ± 0.58	1.89 ± 0.53	<0.001
NLR	4.57 (1.78 ~ 6.65)	2.29 (1.77 ~ 3.25)	0.051
Eosinophils count(x10^9^/L)	0.10 (0.04 ~ 0.21)	0.17 (0.09 ~ 0.27)	0.078
CD15 ^+^ CD274 ^+^ Neutrophil	12.49 (5.03 ~ 23.16)	1.63 (0.91 ~ 4.29)	<0.001

**Abbreviations:** AE-COPD = acute exacerbation of chronic obstructive pulmonary disease; CRP = C-reactive protein; NLR = neutrophil/lymphocyte ratio. FEV_1_ = Forced Expiratory Volume in 1 second; FVC = Forced Vital Capacity.

**Fig 2:**
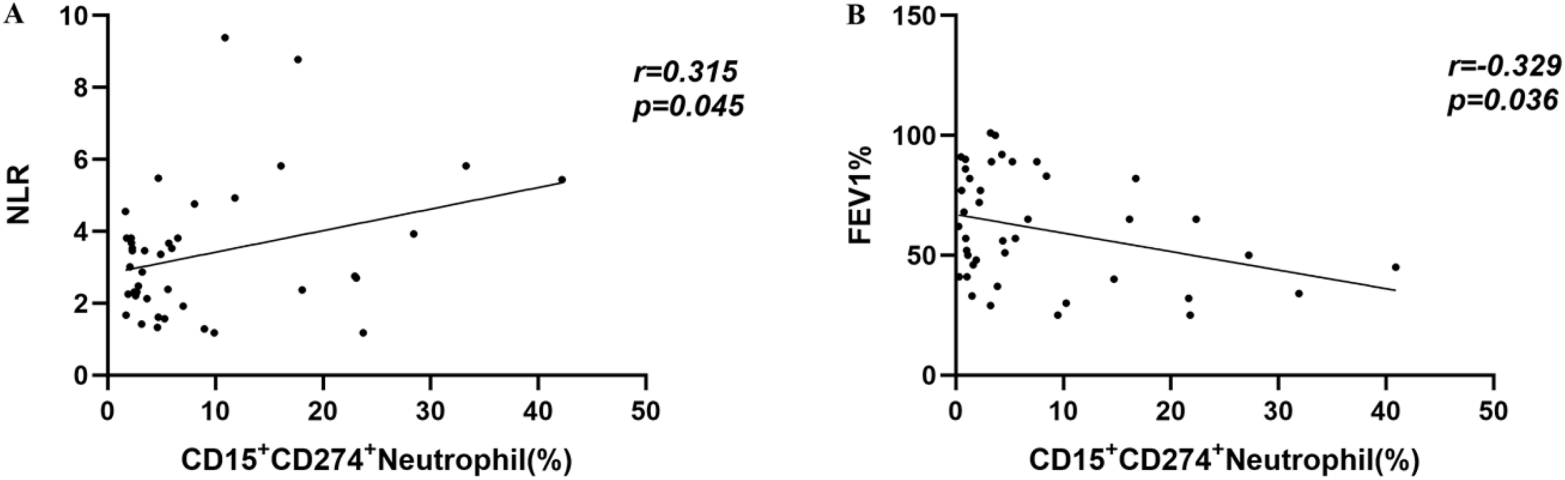
Correlations between the percentage of CD15^+^CD274^+^ neutrophil and NLR (A), and FEV1% (B). **Abbreviations:** NLR= neutrophil/lymphocyte ratio.

Among patients with AECOPD and stable-COPD, the level of NLR was higher and the FEV_1_% was lower as the percentage of CD15^+^CD274^+^ neutrophil increased (Fig 2). Specifically, CD15⁺CD274⁺ neutrophil percentage was positively correlated with NLR (r = 0.315, P = 0.045) and negatively correlated with predicted FEV₁% (r = −0.329, P = 0.036).

**Fig 3:**
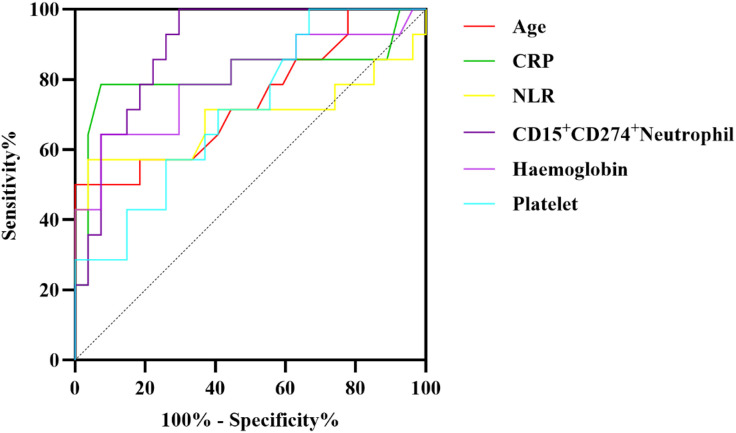
Receiver-operating characteristic curves for age, CRP, NLR, CD15^+^CD274^+^ neutrophil, hemoglobin and platelet for predicting acute exacerbation of COPD. **Abbreviations:** CRP= C-reactive protein; NLR= neutrophil/lymphocyte ratio.

The ROC curves of the age, CRP, NLR, CD15^+^CD274^+^ neutrophil, hemoglobin and platelet were displayed in Fig 3. Table 3 showed that the AUC of the percentage of CD15^+^CD274^+^ neutrophil (0.894; 95% CI, 0.8–0.989) was higher than that of age (0.739; 95% CI, 0.568–0.911), CRP (0.815; 95% CI, 0.642–0.988), NLR (0.688; 95% CI, 0.478–0.897), HGB (0.798; 95% CI, 0.638–0.958) and PLT (0.713; 95% CI, 0.55–0.876).

**Table 3:** ROC Curve Data.

	Age	CRP	NLR	CD15 ^+^ CD274 ^+^ Neutrophil	HGB	PLT
Cutoff value	>71	>9.65	>4.31	>3.273	<127.5	<251
Sensitivity,%	0.571	0.786	0.571	1.000	0.643	1.000
Specificity,%	0.815	0.926	0.963	0.704	1.000	0.333
Positive predictive value,%	0.615	0.846	0.889	0.636	1.000	0.438
Negative predictive value,%	0.786	0.893	0.813	1.000	0.844	1.000
Diagnostic accuracy	0.732	0.878	0.829	0.805	0.878	0.561
Youden’s index	0.386	0.712	0.534	0.704	0.569	0.333
AUC	0.739	0.815	0.688	0.894	0.798	0.713
95%CI	0.568-0.911	0.642-0.988	0.478-0.897	0.8-0.989	0.638-0.958	0.55-0.876
*P value*	0.013	0.001	0.051	<0.001	0.002	0.027

**Abbreviations:** ROC = receiver-operating characteristic; AUC = area under the curve; CI = confidence interval; CRP = C-reactive protein; NLR = neutrophil/lymphocyte ratio; HGB = hemoglobin; PLT = platelet.

The diagnostic accuracy of age, CRP, NLR, CD15^+^CD274^+^ neutrophil, hemoglobin and platelet were 0.732, 0.878, 0.829, 0.805, 0.878 and 0.561. The cutoff value for predicting AECOPD was age > 71, CRP > 9.65, NLR > 4.31, CD15^+^CD274^+^ neutrophil>3.273, HGB < 127.5 and PLT < 251.

**Fig 4:**
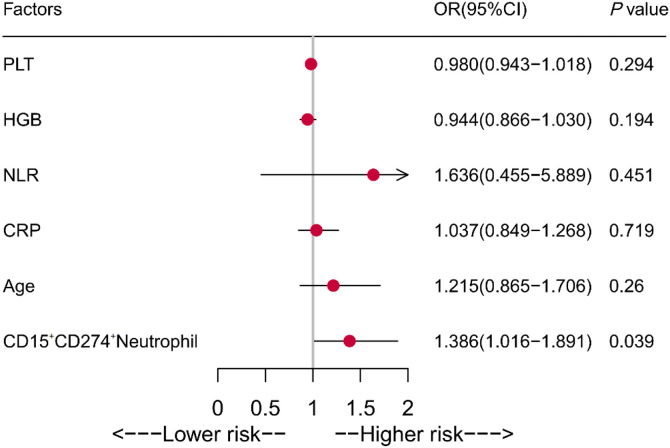
Logistic Regression Analysis of Risk Factors for acute exacerbation of COPD. **Abbreviations:** OR, odds ratio; CI, confidence interval; CRP = C-reactive protein; NLR = neutrophil/lymphocyte ratio; HGB = hemoglobin; PLT = platelet.

In the multivariate logistic regression analysis, candidate variables were selected according to statistically significant intergroup differences between patients with acute exacerbation and those with stable COPD. We incorporated CD15^+^CD274^+^ neutrophil, PLT, HGB, NLR, CRP and age. According to multivariate logistic regression analyses, the percentage of CD15^+^CD274^+^ neutrophil (OR, 1.386; 95% CI, 1.016–1.891; P = 0.039) was independently associated with acute exacerbation of COPD (Fig 4).

## Discussion

Our study demonstrated a significant increased PD-L1 expression on peripheral blood neutrophils in patients with COPD, which was correlated with NLR. Furthermore, elevated PD-L1 expression on neutrophil independently associated with acute exacerbations of COPD.

The innate immune system meticulously modulates the magnitude, nature, and duration of adaptive immune responses through a variety of mechanisms, including antigen presentation, cytokine secretion, co-stimulatory signaling, and regulatory cell activity [17]. The immunopathology of COPD is intrinsically linked to both innate and adaptive inflammatory immune responses. These responses are primarily triggered by the chronic inhalation of cigarette smoke and byproducts from biomass fuel combustion, as well as by intermittent acute exacerbations resulting from infections. This exposure leads to the activation of dendric cells, macrophages, neutrophils and airway epithelial cells. Upon activation, these innate immune cells further facilitate the recruitment and activation of adaptive immune cells by secreting inflammatory mediators [1].

There is no doubt that the imbalance between innate and adaptive immune responses is of great significance yet with predictive values among patients with COPD. The number of macrophages in the small airways increases as the severity of COPD progresses when compared with those of healthy control [18] along with significantly altered macrophage polarization [19]. In addition, there showed retarded maturation of DCs (dendric cell) in COPD, which was also related to disease severity [20]. It has been reported that neutrophils presented with excessively activation during COPD by releasing elastase [21], proteinase-3 [22], matrix metalloproteinase [23], and myeloperoxidase [24], which was directly involved in the pathophysiology of COPD.

The dysregulation of adaptive immunity is also noteworthy and critically involved in COPD prognosis, which showed weak immune response to pathogens due to dysfunction of effector cells and polarization of suppressive T-regulatory cells [25]. The adaptive immune system may also be linked to increased exacerbation risk in the ‘frequent exacerbator’ COPD sub-phenotype, characterized by reduced number of CD4^+^ T central memory and CD8^+^ T effector memory cells [26]. Yusuke Abe demonstrated that lymphocytes from patients with COPD failed to recognize the nontypeable Haemophilus influenzae [27]. In addition, the decreased number of lymphocytes served as an independent risk factor for mortality among COPD patients [28] and was linked to reduced FEV_1_ levels, poor performance in six-minute walk distances, and worse quality life [29]. Therefore, clarifying the crosstalk and its mechanism between innate and adaptive immune responses are of great importance in understanding the progression of COPD.

Neutrophil/lymphocyte ratio (NLR)is a potential laboratory indicator with significant implications in predicting all-cause mortality among patients with COPD [30]. Our research group has previously demonstrated that NLR was more sensitive to predict noninvasive mechanical ventilation failure than traditional inflammatory markers in AECOPD patients [14]. The observed imbalance between the overactivation of neutrophils and the inhibition of T lymphocytes also highlighted the potential link between neutrophils and T cells in COPD patients. The inhibition of lymphocytes is reportedly due to several factors: the increased cortisol levels by intense inflammatory impaired lymphocyte function [31]; reduced lymphocyte count due to elevated interleukin-6 (IL-6) levels [32];as well as commonly noted malnutrition status among COPD patients [33]. In this study, the increased expression of PD-L1 on neutrophils was independently associated with AECOPD, which might be achieved by significantly elevated NLR.

PD-L1/PD-1 axis delivers inhibitory signals which is responsible for retarded T cell activation and proliferation. The expression of PD-L1 has been identified on murine T and B lymphocytes, dendritic cells, macrophages, mesenchymal stem cells, and mast cells derived from bone marrow [34]. Upon ligand binding, PD-1 undergoes phosphorylation at its two intracellular tyrosine residues, and ultimately negatively affect cellular proliferation and cytokine production [35]. PD-L1/PD-1 axis plays crucial role in modulating immune defenses against pathogens in response to both acute and chronic infections. It has been reported that the antagonism of PD-1 induced proliferation of effector T cells during adenovirus infection and resulted in rapid viral clearance [36]. In mice model with persistent viral infection and CD4^+^T cell dysfunction, blocking the PD-L1/PD-1 pathway benefited CD8^+^T cells by restoring proliferation, cytokine secretion, cell-killing ability, and then reducing viral load [37]. In addition, the PD-L1/PD-1 pathway may also be crucial in sustaining chronic bacterial infections. Under exposure to Helicobacter pylori, the expression of PD-L1 significantly increase on human gastric epithelial cells, while anti-PD-L1 antibodies boost T cell proliferation and interleukin-2 (IL-2) production [38].

COPD is characterized by chronic inflamatory responses, which is amplified and further increased during acute exacerbations mostly triggered by infection. Previous study confirmed that patients with COPD showed significantly exaggerated exhaustion of effector T cells due to increased PD-1 expression when compared with healthy controls [39]. Anti-PD-1 rescued lung damage and neutrophilic inflammation in COPD mice models [40]. The current study showed that the levels of PD-L1 on neutrophil were much higher in patients with COPD than that of healthy controls with no significant differences in terms of the comorbidity and cigarette exposure. However, our study found no increase in the NLR of patients with COPD compared to healthy controls, which may be due to the fact that the study was limited by a small sample size of patients with COPD, and also with more patients in a stable condition. Study by Ersin showed that NLR was significantly higher in patients with COPD than controls, but not between patients with stable COPD and with exacerbations [41]. These differences across studies might result from the included sample sizes and the distribution of patients among different groups. A previous review indicated that lung cancer patients with COPD benefit from anti-PD-L1/PD-1 therapy, with improved lung function, such as FeNO (exhaled nitric oxide) levels, FEV_1_, and FVC, partly due to increased PD-1 expression on CD8^+^ cells among patients with COPD [42]. Currently, there is no available immunotherapy for COPD, therefore, further exploration and research in this area may be warranted in the future. Admittedly, the present study cannot confirm that neutrophil PD-L1 serves as a COPD-specific biomarker, and its elevated expression may merely reflect non-specific systemic inflammation. Although our data revealed that neutrophil PD-L1 was negatively correlated with predicted FEV₁ and positively correlated with NLR in COPD patients, further mechanistic experiments are still required to validate whether neutrophil PD-L1 is specific to COPD. Moreover, the optimal cutoff value for the percentage of CD15⁺CD274⁺ neutrophils was determined solely based on ROC analysis of the current single-center cohort. Therefore, this threshold requires prospective external validation using an independent patient cohort before its clinical predictive value and practical applicability can be definitively established.

In current study, compared with stable COPD, patients with AECOPD exhibited higher NLR and we further confirmed that neutrophil PD-L1 expression was positively correlated with NLR. However, no significant NLR differences were observed between the AECOPD and stable groups, possibly due to the limited sample size. It might suggest that the sub-phenotype of neutrophils, rather than its total number, was responsible for the alteration of NLR. Our results were also consistent with those findings by previous study that NLR was an independent risk factor for patients with COPD with exacerbations, but the potential mechanism remains poorly understood [43]. This study proposes the critical involvement of the PD-L1/PD-1 axis in AECOPD immune dysregulation. These findings were further confirmed by significant correlation between the expression of PD-L1 on neutrophil and FEV1%, which was also conformed with previously reported correlation between NLR and FEV1% [5].

In our study, the cutoff value of NLR for predicting AECOPD is 4.31, which was similar with other studies that found NLR values over 3.29 [44] and 3.34 [45] were able to predict AECOPD. PD-L1 expression was the only statistically significant predictor of AECOPD in regression analysis, exhibiting the highest area under ROC curve in current study. Nevertheless, given that our investigation is exploratory with limited AECOPD cases, the established logistic regression model is accompanied by a prominent risk of overfitting. We found that a PD-L1 cutoff value of 3.273 is independently associated with the occurrence of AECOPD. Considering the observed positive correlation between PD-L1 expression and NLR, it is necessary to conduct further research to determine whether PD-L1 can induce modifications in NLR, thereby affecting the prognosis of patients with COPD.

### Limitation

This study had certain limitations. First, the small overall sample size limited statistical power and introduced risk of model overfitting. The wide AUC 95% CI reflected large uncertainty, so the predictive value of neutrophil PD-L1 requires careful interpretation. Secondly, some other outcomes, such as long-term control of symptoms, activity of daily life, and mortality also remain unclarified due to small sample sizes and short-term follow-up. Thirdly, this study did not confirm direct contact between neutrophil PD-L1 and lymphocytes, although increased neutrophil PD-L1 expression was associated with lymphocytes; Lastly, multiple statistical comparisons were performed in this study. Given its exploratory nature, we did not apply Bonferroni correction for multiple testing, which may increase the risk of false-positive findings. Larger-scale, multicenter cohorts and in-depth mechanistic experiments are urgently needed to further verify its reliability and fully define its practical clinical utility.

## Conclusion

Within this pilot patient cohort, peripheral blood neutrophil PD-L1 expression was elevated in patients with AECOPD and independently associated with exacerbation status. Larger multicenter prospective cohorts paired with mechanistic laboratory validation are required to define its full clinical diagnostic utility.

## Abbreviations

PD-1-PD-L1: programmed death receptor 1 and its ligand
COPD: chronic obstructive pulmonary disease
CRP: C-reactive protein
NLR: neutrophil/lymphocyte ratio
AE-COPD: acute exacerbation of chronic obstructive pulmonary disease
FEV1: Forced Expiratory Volume in 1 second
FVC: Forced Vital Capacity
ROC: receiver-operating characteristic
AUC: area under the curve
CI: confidence interval
OR: odds ratio
HGB: hemoglobin
PLT: platelet.
